# Supplementary material for: The Leaf Microbiome of Tobacco Plants across Eight Chinese Provinces
Source: Microorganisms. 2022 Feb 16;10(2):450. doi: 10.3390/microorganisms10020450 (PMC8878116; doi:10.3390/microorganisms10020450)
Supplement: Supplementary file 1 [file microorganisms-10-00450-s001.zip › microorganisms-1566827-supplementary.pdf]

The leaf microbiome of tobacco plants across 8 Chinese Provinces

Haiyang Hu[1†*], Yunli Liu[1†], Yiqun Huang[1], Zhan Zhang[2], Hongzhi Tang[1]

1 State Key Laboratory of Microbial Metabolism, School of Life Sciences, Biotechnology, Shanghai Jiao Tong University, Shanghai 200240, China; liuyunli@sjtu.edu.cn (Y.L.); natsumoon@outlook.com (Y.H.); tanghongzhi@sjtu.edu.cn (H.T.)

2 China Tobacco Henan Industrial Co., Ltd., Zhengzhou 450000, China; zhangzhan2059729@126.com

* Correspondence: huhaiyang@sjtu.edu.cn; Tel.: +86-21-34204066; Fax: +86-21-34206723

† These authors contributed equally

‡ Zhang Zhan is a visiting researcher in Shanghai Jiaotong University. He worked here from 8-October to 29 December in 2020 and from 1 Apr to 30 Jun 2021.

Table S1. The genera with top 13 relative abundances among all samples.

No.	Genus	Relative abundance
1	*Mastigocoleus*	9.40%
2	*Pseudomonas*	9.20%
3	*Methylobacterium*	7.90%
4	*Sphingomonas*	5.90%
5	*Acinetobacter*	5.90%
6	*Salmonella*	4.30%
7	*Iphinoe*	1.40%
8	*Siccibacter*	1.10%
9	*Enterobacter*	6.10%
10	*Pantoea*	5.70%
11	*Leclercia*	4.60%
12	*Atlantibacter*	3.40%
13	*Bacillus*	1.10%

Table S2. Values for diversity indices including Chao1, ACE, Shannon, and Simpson.

No.	Sample names	Abbreviation	Chao1	ACE	Shannon	Simpson
1	Mangdui	YNMD-1	496.38	540.66	5.43	0.91404
2		YNMD-2	334.97	348.1	4.66	0.879039
3		YNMD-3	462.02	482.6	6.89	0.977228
4	Mengligong	YNMLG-1	436.24	454.06	6.24	0.965717
5		YNMLG-2	434.51	464.38	6	0.948951
6		YNMLG-3	420.71	436.13	6.54	0.977544
7	Menglijiaojid	YNJD-1	502.11	531.2	6.51	0.96351
8		YNJD-2	262.39	266	5.57	0.940027

9		YNJD-3	397.14	424.89	5.63	0.931951
10		YNJD-4	657.01	719.48	7.17	0.980499
11	Qujing	YNQJ	444.97	480.36	5.79	0.947153
12		YNWS-1	723.4	722.88	7.41	0.987752
13	Wenshan	YNWS-2	591.31	631.2	7.22	0.985375
14		YNWS-3	436.24	462.35	6.44	0.973301
15		YNCX-1	296.41	314.21	4.53	0.899527
16	Chuxiong	YNCX-2	391.03	426.02	6.16	0.969601
17		YNCX-3	448.15	472.89	6.16	0.956488
18	Xindian	YNXD-1	436	468.93	6.34	0.975027

19		YNXD-2	410.86	421.92	6.89	0.984097
20		YNXD-3	335.16	356.52	4.64	0.896422
21	Lijiang	YNLJ-1	400.12	423.49	6.65	0.980293
22		YNLJ-2	479	479	7.1	0.978974
23		YNHP-1	719.08	768.87	7.42	0.984453
24	Huaping	YNHP-2	504.19	543.05	6.63	0.977342
25		YNHP-3	343	343	6.26	0.968324
26		HeNZW-1	380.24	413.29	4.72	0.863809
27	Zhangwu	HeNZW-2	333.65	347.01	4.79	0.88234
28		HeNZW-3	284.12	317.46	4.61	0.892561

29		HeNPDS-1	359.09	372.8	6.57	0.980296
30	Pingdingshan	HeNPDS-2	377.14	405.89	6.3	0.970262
31		HeNPDS-3	570.03	598.2	7.3	0.98653
32	Luoyang	HeNLY-1	505.88	516.13	6.2	0.955718
33		HeNLY-2	475.64	499.52	5.68	0.93912
34	Zhuyang	HeN-ZY	178.4	190.34	2.84	0.742921
35	Sanmenxia	HeNSMX	368.04	383.34	4.35	0.844246
36	Xuchang	HeNXC-1	438.5	463.54	6.15	0.957591
37		HeNXC-2	414.02	415.17	7.11	0.985082
38	Shigang	HeNSG-1	366.02	397.39	5.37	0.939487

39		HeNSG-2	459.79	491.76	5.65	0.933176
40	Xiangcheng	HeNXC	391.75	419.94	5.82	0.958948
41	Chongqing	CQ-1	339.01	363.29	5.56	0.951285
42		CQ-2	526.28	555.93	6.44	0.963472
43	Yangshi	HuNYS-1	614.25	641.34	6.78	0.975255
44		HuNYS-2	399.76	423.5	5.58	0.920859
45		HuNYS-3	287.38	304.44	5.86	0.963191
46		HuNYS-4	226.51	234.31	4.12	0.861432
47		HuNYS-5	530.29	573.24	6.82	0.974697
48		HuNYS-6	575.05	616.85	6.77	0.970914

49	Bijie	GZBJ-1	508.77	541.8	6.04	0.962006
50		GZBJ-2	482.45	510.35	6.51	0.975451
51		GZBJ-3	284.12	304.11	3.92	0.800097
52		GZBJ-4	531.93	561.92	6.82	0.98226
53		GZBJ-5	393.85	396.19	5.1	0.908417
54		GZBJ-6	360.07	353.33	4.84	0.90591
55	Weining	GZWN-1	372	372	6.77	0.974709
56		GZWN-2	481.06	506.21	6.73	0.979132
57	Qianxinan	GZQXN-1	381.91	395.07	6.54	0.970156
58		GZQXN-2	262.78	285.57	4.13	0.842303

59		GZQXN-3	259.2	271.08	4.96	0.930895
60		GZQXN-4	409.46	443.3	6.13	0.967219
61		SCPZH-1	498.74	507.64	5.76	0.931751
62	Panzhihua	SCPZH-2	418.07	446.04	5.33	0.909534
63		SCPZH-3	367.5	395.64	5.31	0.926667
64		HLJMDJ-1	384.74	407.39	6.11	0.966191
65		HLJMDJ-2	407.54	419.33	6.88	0.983132
66	Mudanjiang	HLJMDJ-3	548.8	578.12	6.43	0.961567
67		HLJMDJ-4	533.14	559.49	7.11	0.984816
68		HLJMDJ-5	421.91	422.08	6.1	0.968276

69		FJSM-1	667.31	714.31	7	0.975688
70	Sanming	FJSM-2	553.02	543.94	6.78	0.975188
71		FJSM-3	429.74	440.72	6.75	0.976319
72		FJSW-2	421	421	7.16	0.983821
73		FJSW-1	479.71	491.17	7.1	0.984062
74		FJSW-3	528.62	511.23	6.62	0.972505
75	Shaowu	FJSW-5	352.37	359.01	5.87	0.952252
76		FJSW-6	332.01	332.75	6.28	0.970516
77		FJSW-7	331.88	343.71	5.43	0.943821
78		FJSW-4	514.94	526.67	6.77	0.971637

Table S3. Lists of the core microbial communities determined for each province.

Province	Core microbial community
Yunnan	*Mastigocoleus testarum*
	Atlantibacter hermannii
	Salmonella enterica
	Xanthomonas campestris
	Leclercia adecarboxylata
	Enterobacter soli
	Pseudomonas oryzihabitans、*Pseudomonas straminea*
	Methylobacterium goesingense
	Pantoea agglomerans
	Sphingomonas roseiflava; Sphingomonas aurantiaca; Sphingomonas aerolata
	Ochrobactrum anthropi
	Agrobacterium larrymoorei
Guizhou	*Mastigocoleus testarum*
	Atlantibacter hermannii
	Salmonella enterica
	Leclercia adecarboxylata
	Enterobacter soli
	Pantoea agglomerans
	Pseudomonas oryzihabitans
	Acinetobacter johnsonii
	Methylobacterium goesingense
	Sphingomonas aurantiaca
Fujian	*Salmonella enterica*
	Bordetella petrii; *Bordetella hinzii*
	Atlantibacter hermannii

	Franconibacter helveticus
	Leclercia adecarboxylata
	Enterobacter soli; Enterobacter xiangfangensis; Enterobacter cancerogenus
	Pseudomonas oryzihabitans; Pseudomonas straminea; Pseudomonas azotifigens
	Acinetobacter baumannii
	Pantoea agglomerans; Pantoea intestinalis
	Methylobacterium goesingense; Methylobacterium hispanicum
	Sphingomonas roseiflava; Sphingomonas phyllosphaerae
	Beijerinckia fluminensis
	Paracoccus yeei
	Pigmentiphaga daeguensis
	Ochrobactrum pseudintermedium
Hunan	*Mastigocoleus testarum*
	Siccibacter colletis
	Atlantibacter hermannii
	Klebsiella
	Salmonella enterica
	Leclercia adecarboxylata
	Enterobacter soli; Enterobacter xiangfangensis
	Pseudomonas oryzihabitans; Pseudomonas straminea; Pseudomonas parafulva
	Acinetobacter johnsonii
	Pantoea agglomerans; Pantoea dispersa
	Sphingomonas roseiflava; Sphingomonas phyllosphaerae; Sphingomonas sanguinis
	Methylobacterium hispanicum
Sichuan	*Mastigocoleus testarum*

	Atlantibacter hermannii
	Salmonella enterica
	Leclercia adecarboxylata
	Enterobacter soli
	Pseudomonas oryzihabitans; Pseudomonas parafulva
	Acinetobacter johnsonii
	Methylobacterium goesingense
	Ochrobactrum anthropi; Ochrobactrum lupini
	Pantoea agglomerans; Pantoea dispersa
	Sphingomonas roseiflava
	Mastigocoleus testarum
	Atlantibacter hermannii
	Citrobacter murliniae
	Aurantimonas phyllosphaerae
	Arthrospira platensis
	Chroococcidiopsis thermalis
	Salmonella enterica
	Kluyvera intermedia
	Stenotrophomonas maltophilia
Chongqing	*Serratia proteamaculans*
	Leclercia adecarboxylata
	Enterobacter soli; Enterobacter cancerogenus
	Pseudomonas oryzihabitans; Pseudomonas punonensis
	Pantoea agglomerans
	Methylobacterium goesingense
	Sphingomonas roseiflava; Sphingomonas aurantiaca; Sphingomonas phyllosphaerae; Sphingomonas cynarae; Sphingomonas aerolata; Sphingomonas yunnanensis
	Ochrobactrum anthropi; Ochrobactrum lupini

	Acinetobacter guillouiae; Acinetobacter bereziniae
Henan	*Mastigocoleus testarum*<br>*Salmonella enterica*<br>*Leclercia adecarboxylata*<br>*Pseudomonas oryzihabitans*<br>*Pantoea agglomerans*<br>*Methylobacterium goesingense; Methylobacterium hispanicum;*<br>*Methylobacterium brachiatum; Methylobacterium mesophilicum*<br>*Enterobacter xiangfangensis*
Heilongjiang	*Mastigocoleus testarum*<br>*Escherichia vulneris*<br>*Franconibacter helveticus*<br>*Atlantibacter hermannii*<br>*Stenotrophomonas maltophilia*<br>*Salmonella enterica*<br>*Leclercia adecarboxylata*<br>*Enterobacter soli; Enterobacter xiangfangensis; Enterobacter cancerogenus*<br>*Pantoea agglomerans; Pantoea intestinalis; Pantoea dispersa*<br>*Pseudomonas oryzihabitans*<br>*Methylobacterium goesingense; Methylobacterium brachiatum;*<br>*Methylobacterium mesophilicum*<br>*Sphingomonas roseiflava*<br>*Beijerinckia fluminensis*<br>*Agrobacterium larrymoorei*

Table S4. Lists of beneficial genera uniquely present in particular provinces.

Province	Genus	Function	Relative abundance
Yunnan	*Ochrobactrum*	Heavy metals detoxification, and nitrogen fixation	24.91%
	Agrobacterium	Degradation of pesticides	
Guizhou	*Acinetobacter*	Degradation of PAHs and nicotine, heavy metals detoxification	23.63%
Fujian	*Franconibacter*	Degradation of alkane	23.02%
	Beijerinckia	Nitrogen fixation	
	Paracoccus	Degradation of formaldehyde	
	Acinetobacter	Degradation of PAHs and nicotine, heavy metals detoxification	
	Pigmentiphaga	Degradation of PAHs, pesticides and nicotine	
	Ochrobactrum	Degradation of PAHs	
Hunan	*Acinetobacter*	Degradation of PAHs and nicotine, heavy metals detoxification	19.11%
Sichuan	*Acinetobacter*	Degradation of PAHs and nicotine, heavy metals detoxification	33.23%
	Ochrobactrum	Degradation of pesticides	
Chongqing	*Acinetobacter*	Degradation of PAHs and nicotine, heavy metals detoxification	34.98%
	Ochrobactrum	Degradation of pesticides	
	Serratia	Heavy metals detoxification,nitrogen fixation	
Heilongjiang	*Agrobacterium*	Degradation of pesticides	39.80%
	Beijerinckia	Nitrogen fixation	

Table S5. Information about environmental factors (longitude, latitude, annual average temperature, annual precipitation, and altitude) for each sampling location.

No.	Sample	Longitude (E)	Latitude (N)	Annual average temperature (℃)	Annual precipitation (mm)	Altitude (m)
1	YNJD-1	99.2	23.2	17.7	1747.2	1502.4
2	YNJD-2	99.2	23.2	17.7	1747.2	1502.4
3	YNJD-3	99.2	23.2	17.7	1747.2	1502.4
4	YNJD-4	99.2	23.2	17.7	1747.2	1502.4
5	YNMD-1	99.4	23.9	17.8	1148.9	1695.0
6	YNMD-2	99.4	23.9	17.8	1148.9	1695.0
7	YNMD-3	99.4	23.9	17.8	1148.9	1695.0
8	YNMLG-1	99.4	23.9	17.8	1148.9	1695.0
9	YNMLG-2	99.4	23.9	17.8	1148.9	1695.0
10	YNMLG-3	99.4	23.9	17.8	1148.9	1695.0
11	YNLJ-1	100.2	26.9	12.9	980.3	2393.2
12	YNLJ-2	100.2	26.9	12.9	980.3	2393.2
13	YNHP-1	101.3	26.6	19.6	1087.8	1244.8

14	YNHP-2	101.3	26.6	19.6	1087.8	1244.8
15	YNHP-3	101.3	26.6	19.6	1087.8	1244.8
16	YNCX-1	101.3	25.2	14.8	843.2	1859.0
17	YNCX-2	101.3	25.2	14.8	843.2	1859.0
18	YNCX-3	101.3	25.2	14.8	843.2	1859.0
19	SCPZH-1	101.9	27.1	20.9	838.7	1156.0
20	SCPZH-2	101.9	27.1	20.9	838.7	1156.0
21	SCPZH-3	101.9	27.1	20.9	838.7	1156.0
22	YNXD-1	102.9	26.9	11.8	674.6	1949.5
23	YNXD-2	102.9	26.9	11.8	674.6	1949.5
24	YNXD-3	102.9	26.9	11.8	674.6	1949.5
25	YNQJ	103.5	25.5	15.1	944.8	1898.7
26	GZWN-1	104.1	26.9	10.8	859.4	2237.5
27	GZWN-2	104.1	26.9	10.8	859.4	2237.5
28	YNWS-1	104.4	23.0	18.4	974.6	1271.6
29	YNWS-2	104.4	23.0	18.4	974.6	1271.6
30	YNWS-3	104.4	23.0	18.4	974.6	1271.6

31	GZQXN-4	104.9	25.1	18.3	1476.7	1626.0
32	GZQXN-1	104.9	25.5	13.9	1353.8	1626.0
33	GZQXN-2	104.9	25.5	13.9	1353.8	1626.0
34	GZQXN-3	104.9	25.5	13.9	1353.8	1626.0
35	GZBJ-6	105.3	27.3	13.0	865.9	1510.6
36	GZBJ-5	105.4	26.8	13.0	865.9	1510.6
37	GZBJ-1	105.6	27.1	12.1	1085.4	1510.6
38	GZBJ-2	105.6	27.1	12.1	1085.4	1510.6
39	GZBJ-3	105.6	27.1	12.1	1085.4	1510.6
40	GZBJ-4	105.7	27.0	13.0	865.9	1510.6
41	CQ-1	108.8	29.5	17.5	1126.4	259.1
42	CQ-2	108.8	29.5	15.7	1172.8	259.1
43	HeN-ZY	110.7	34.3	13.5	598.7	662.0
44	HeNSMX	111.2	34.8	14.4	516.6	410.1
45	HeNLY-1	111.6	34.4	13.9	568.4	154.5
46	HeNLY-2	111.6	34.4	13.9	568.4	154.5
47	HeNSG-1	111.8	32.9	15.2	793.4	129.8

48	HeNSG-2	111.8	32.9	15.2	793.4	129.8
49	HeNZW-1	111.8	34.4	14.7	630.1	154.5
50	HeNZW-2	111.8	34.4	14.7	630.1	154.5
51	HeNZW-3	111.8	34.4	14.7	630.1	154.5
52	HuNYS-1	112.8	26.0	18.4	1503.4	184.9
53	HuNYS-2	112.8	26.0	18.4	1503.4	184.9
54	HuNYS-3	112.8	26.0	18.4	1503.4	184.9
55	HuNYS-4	112.8	26.0	18.4	1503.4	184.9
56	HuNYS-5	112.8	26.0	18.4	1503.4	184.9
57	HuNYS-6	112.8	26.0	18.4	1503.4	184.9
58	HeNPDS-1	113.2	33.8	14.8	783.4	84.7
59	HeNPDS-2	113.2	33.8	14.8	783.4	84.7
60	HeNPDS-3	113.2	33.8	14.8	783.4	84.7
61	HeNXC	113.5	33.9	14.9	754.3	86.0
62	HeNXC-1	113.6	33.9	14.6	733.5	71.9
63	HeNXC-2	113.6	33.9	14.6	733.5	71.9
64	FJSW-2	117.5	27.3	18.2	1840.9	191.5

65	FJSW-1	117.5	27.3	18.2	1840.9	191.5
66	FJSW-3	117.5	27.3	18.2	1840.9	191.5
67	FJSW-5	117.5	27.3	18.2	1840.9	191.5
68	FJSW-6	117.5	27.3	18.2	1840.9	191.5
69	FJSW-7	117.5	27.3	18.2	1840.9	191.5
70	FJSW-4	117.5	27.3	18.2	1840.9	191.5
71	FJSM-1	117.6	26.3	19.6	1665.2	421.0
72	FJSM-2	117.6	26.3	19.6	1665.2	421.0
73	FJSM-3	117.6	26.3	19.6	1665.2	421.0
74	HLJMDJ-1	129.6	44.6	4.8	561.2	241.4
75	HLJMDJ-2	129.6	44.6	4.8	561.2	241.4
76	HLJMDJ-3	129.6	44.6	4.8	561.2	241.4
77	HLJMDJ-4	129.6	44.6	4.8	561.2	241.4
78	HLJMDJ-5	129.6	44.6	4.8	561.2	241.4

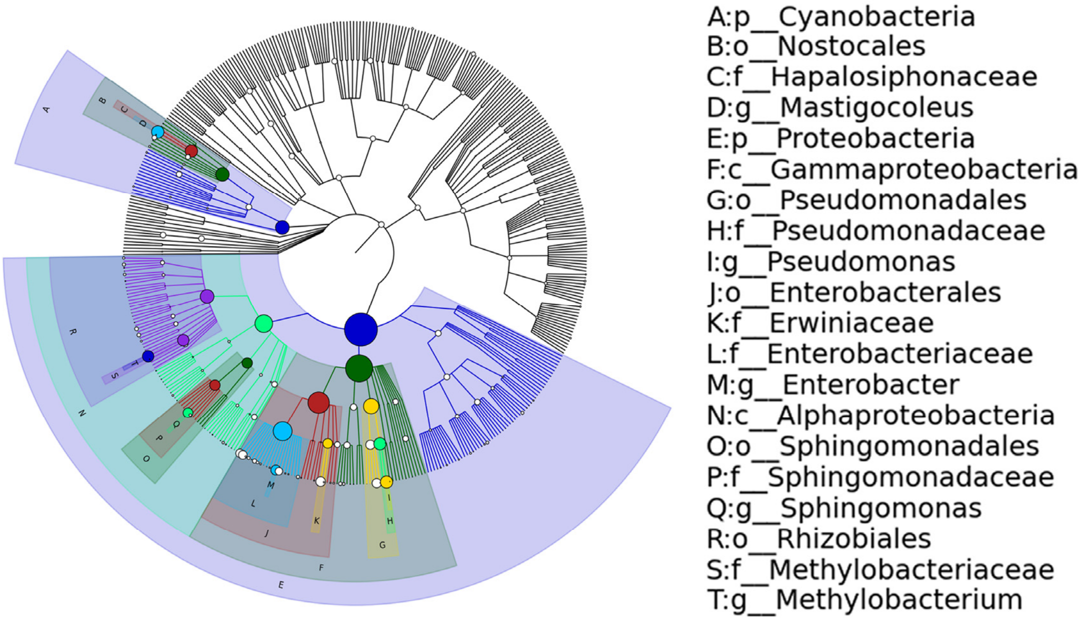

Figure S1. Classification tree for all samples, visualized using GraPhlAn.

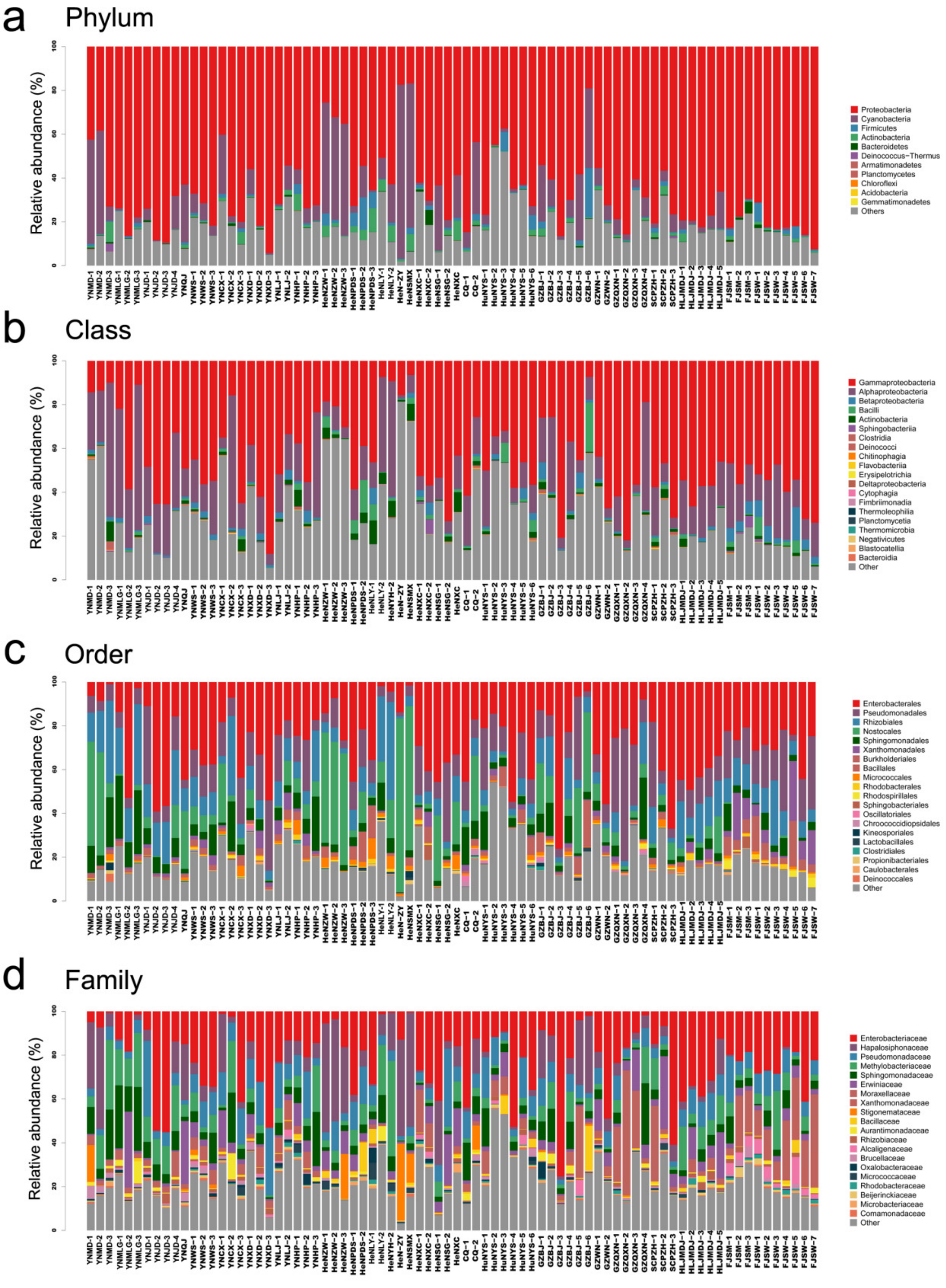

Figure S2. The community compositions at different levels. **(A)** The community compositions at the phylum-level. **(B)** The community compositions at the class-level. **(C)** The community compositions at the order-level. **(D)** The community compositions at the family-level.

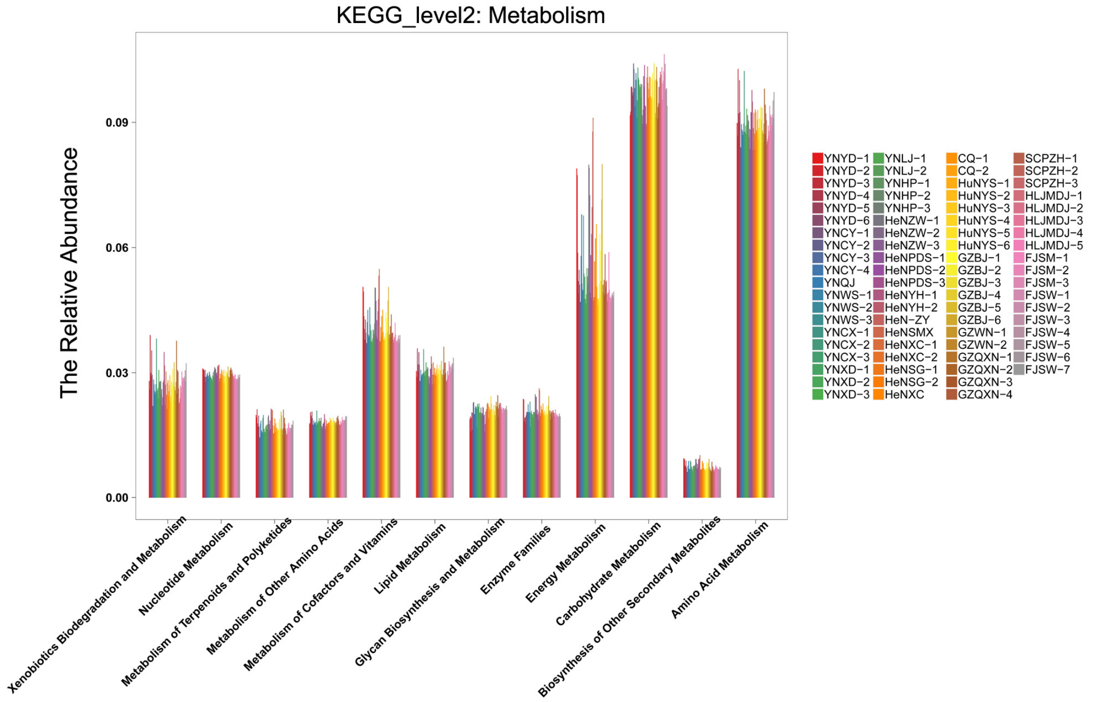

Figure S3. PICRUSt predicted the KEGG-level abundance distributions.

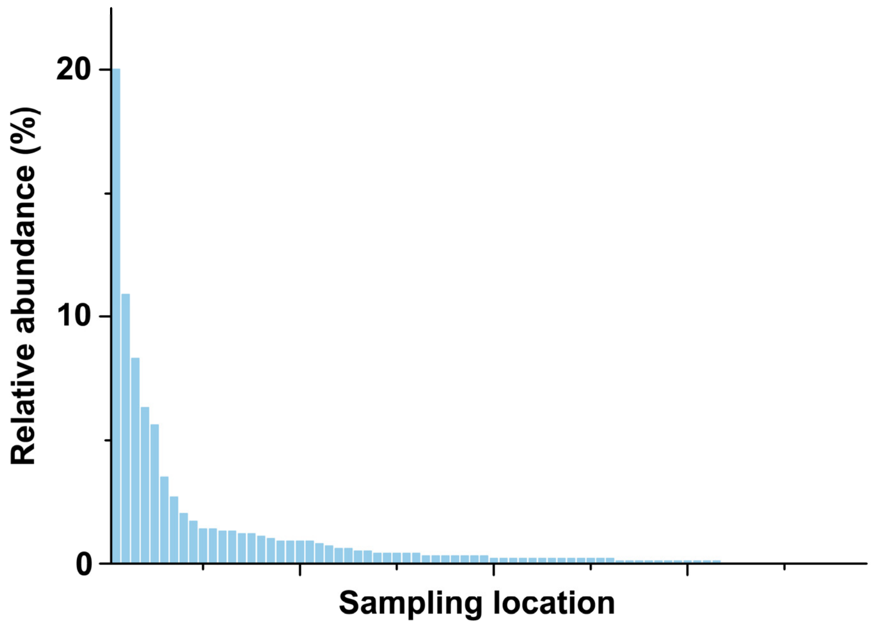

Figure S4. Relative abundance of *Bacillus* of each sample.

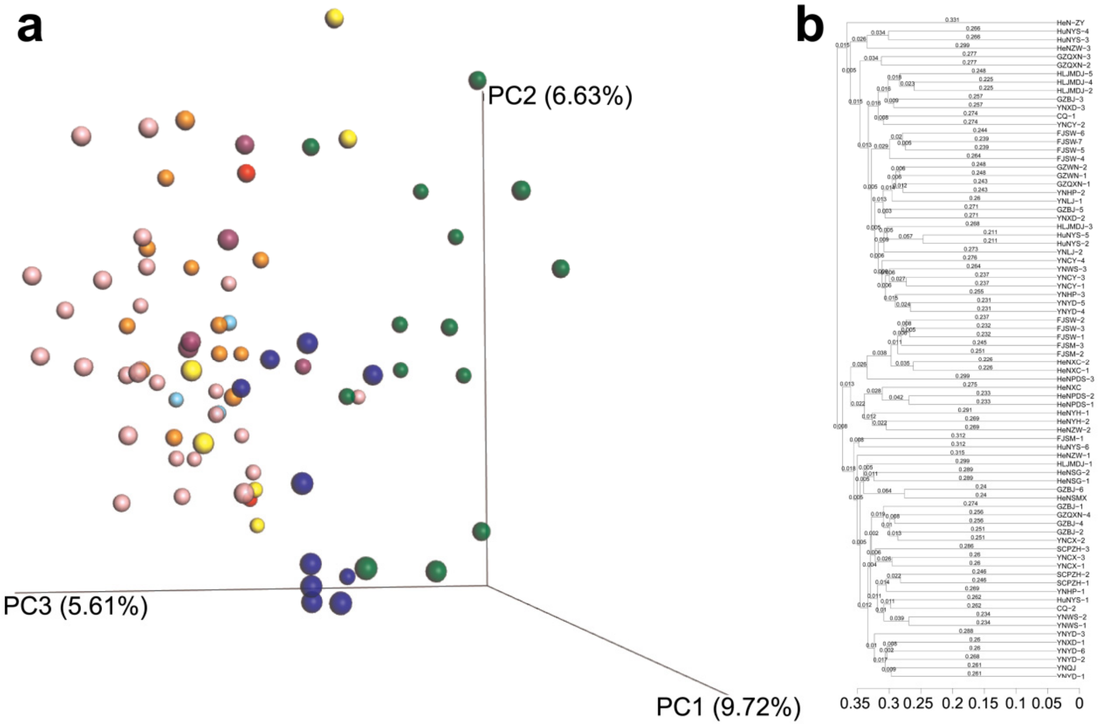

Figure S5. The results of PcoA analysis of unweighted Unifrac distance and unweighted pair-group method with arithmetic means.

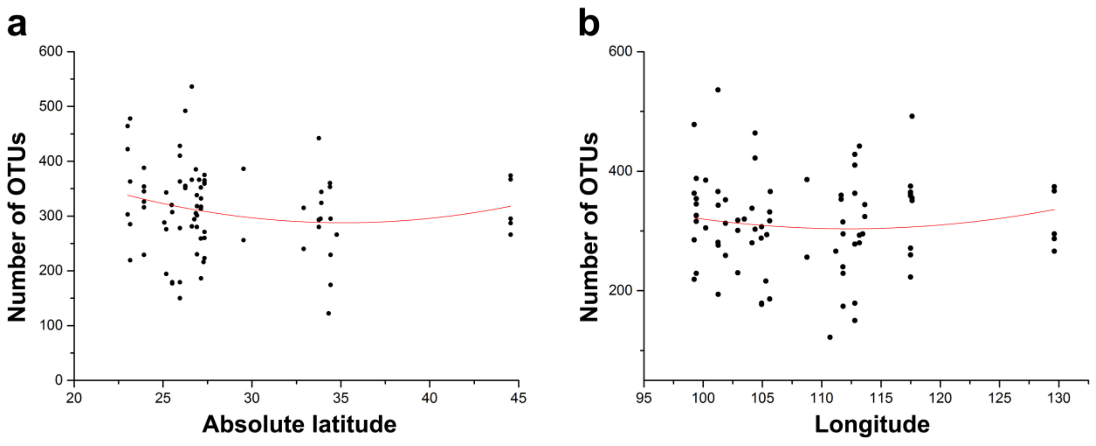

Figure S6. Distribution of microbial diversity. **(A)** Latitudinal distribution of microbial diversity. **(B)** longitudinal distribution of microbial diversity.
